# Supplementary material for: Enterotoxigenic Escherichia coli heat-labile toxin drives enteropathic changes in small intestinal epithelia
Source: Nat Commun. 2022 Nov 12;13:6886. doi: 10.1038/s41467-022-34687-7 (PMC9653437; doi:10.1038/s41467-022-34687-7)
Supplement: Supplementary file 1 — Supplementary Information [file 41467_2022_34687_MOESM1_ESM.pdf]

Supplementary Figures

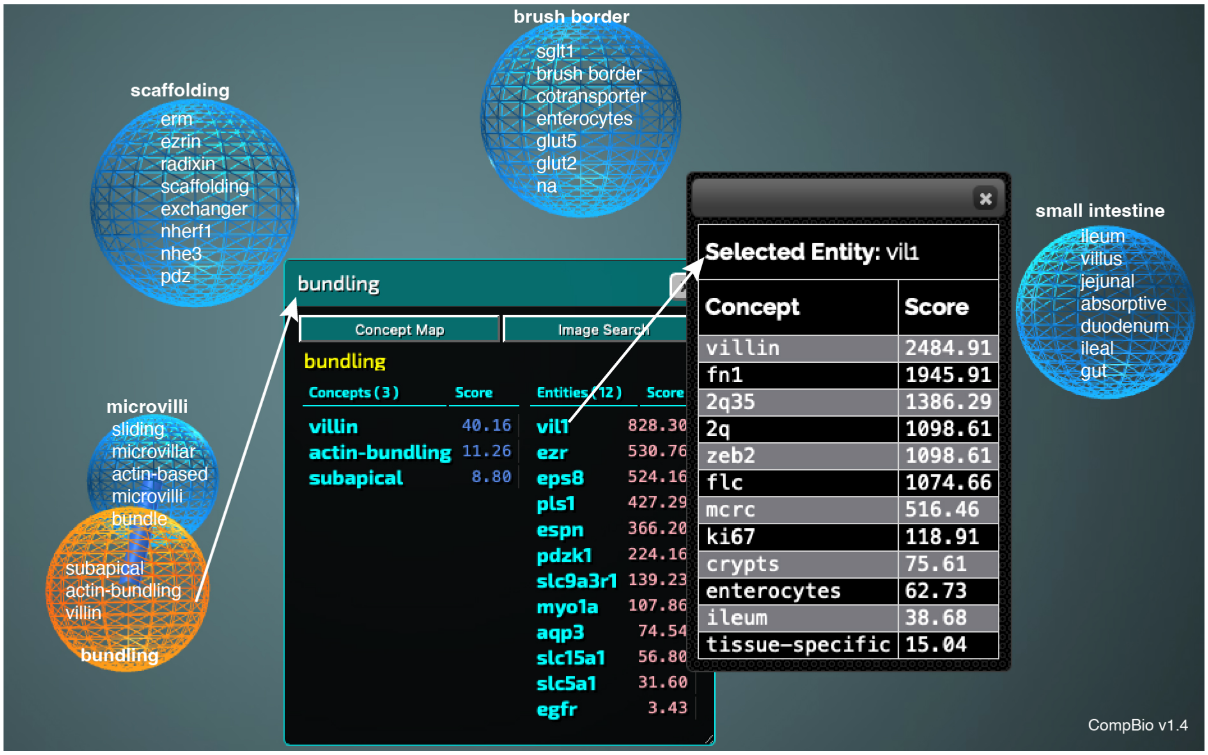

Supplementary figure 1. image from representative CombBio (v1.4) analysis of differential gene expression in LT-treated enteroids compared to untreated controls, highlighting themes (spheres) related to biogenesis and function of small intestinal microvilli. Concepts linked within each theme are listed on the corresponding sphere.

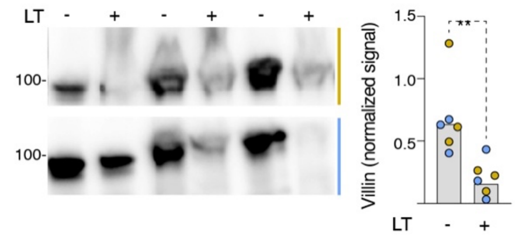

Supplementary figure 2. Villin production is decreased in LT-treated small intestinal enteroids. Shown are villin immunoblot signals in membrane preparations from 2 independent experiments (coded by color), each with 3 technical replicates. LT+ indicates overnight ~16 h treatment of ileal enteroids with heat-labile toxin [100 μg/ml]. Graph summarizes villin immunoblot signals normalized to total protein. (**p=0.0043 Mann Whitney, two-tailed comparisons).

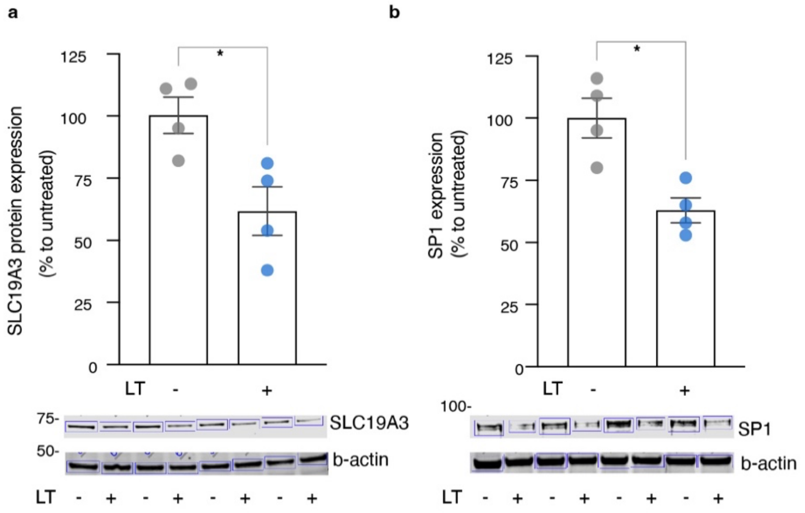

Supplementary figure 3. Impact of LT on expression of SLC19A3 and its associated SP1 transcription factor. Graphs depict summary of immunoblot band intensities of SLC19A3 (**a**) and the SP1 transcription factor (**b**) generated on probing LT-treated small intestinal enteroids (235D) relative to untreated controls. *<0.05 by Mann Whitney two-tailed analysis. Bars reflect geometric mean data ± SEM. The corresponding immunoblots from 4 independent experimental replicates are shown below each graph.

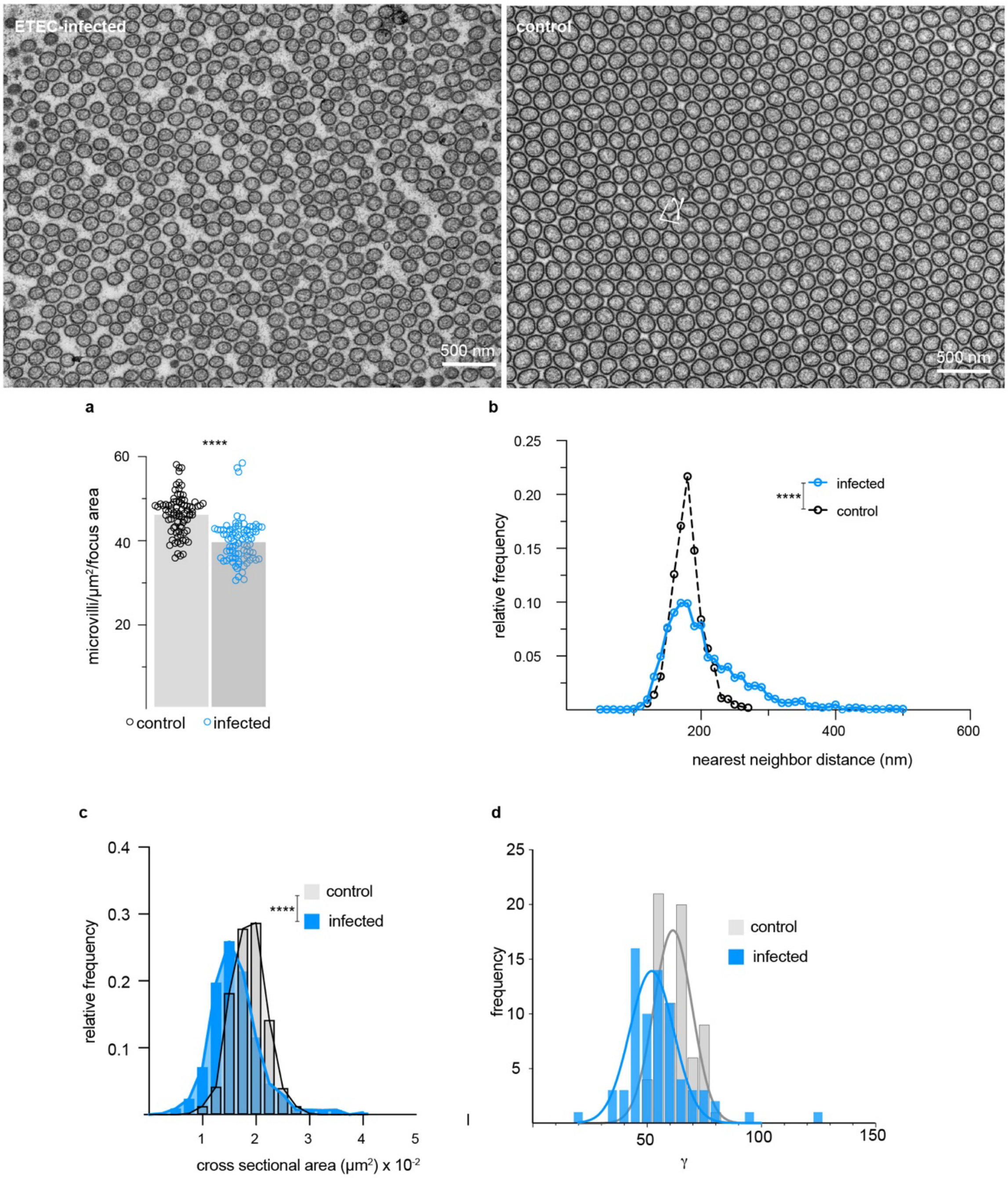

Supplementary figure 4. ETEC perturbs the ordered array structure of intestinal microvilli. Shown at top are representative TEM cross sections of infected and control mice (n=5) in each group. **a.** density of microvilli/µm^2 (n=83 areas in infected and control), bars represent geometric mean data. **b.** distance between centers of adjacent microvilli (n=1001 control, n=2278 infected) **c.** cross sectional area of microvilli (n=550 control, n=1559 infected). In **a-c** **** represents $p<0.0001$ Mann-Whitney (2-tailed) comparisons of groups. **d.** γ angle distributions of microvilli from control and ETEC-infected mice.

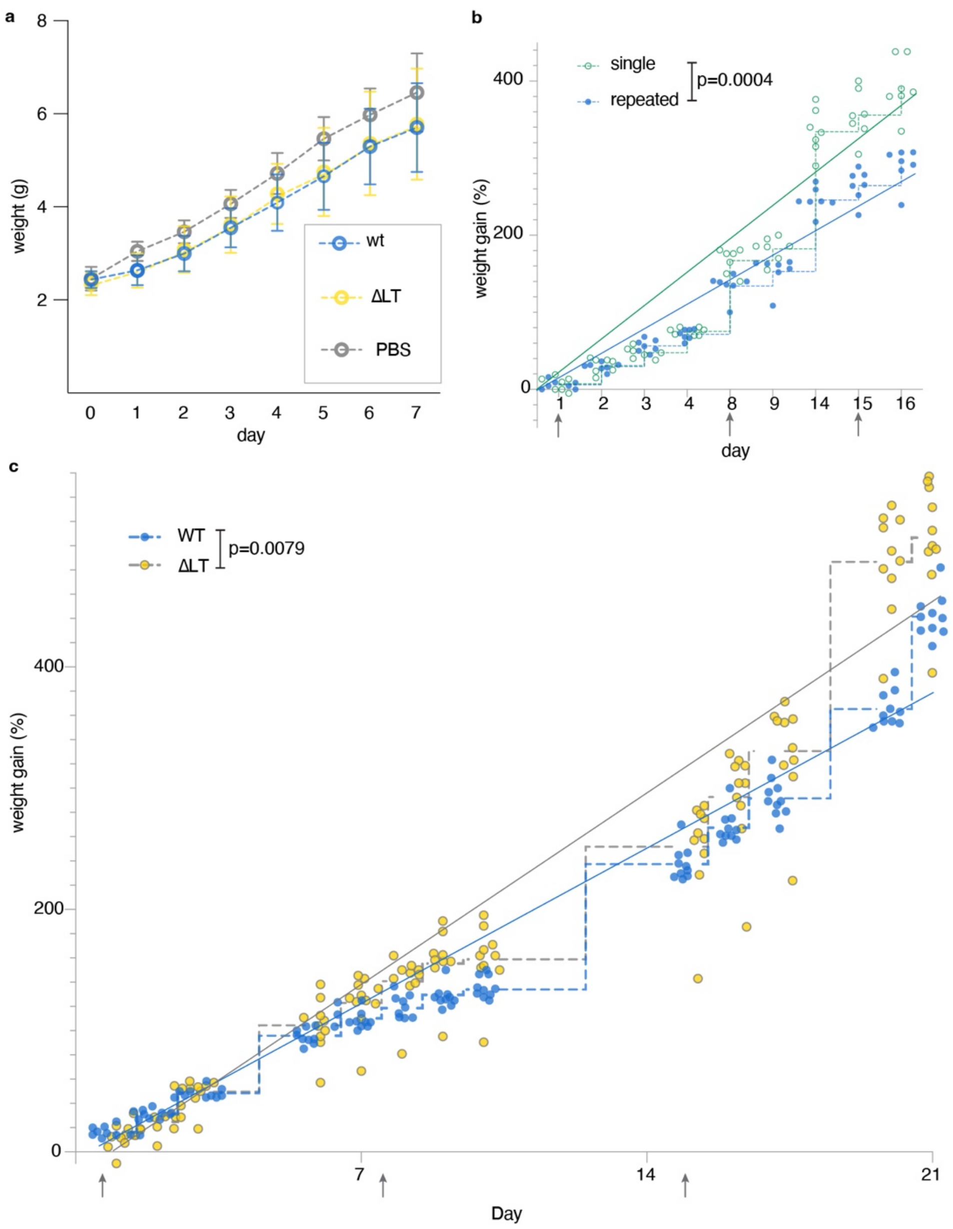

Supplementary figure 5. growth kinetics following challenge of suckling mice with ETEC

a. Short-term growth kinetics of mice singly challenged with wild type toxigenic ETEC H10407 (WT), LT mutant (*ΔeltA,* jf571), vs sham challenge (PBS) (n=24/group). data points reflect mean ± sd. **b.** Repeated vs single challenge of mice with wild type toxigenic ETEC H10407 (wt) (n=7 mice/group); dashed lines connect geometric mean values. Solid lines = linear regression. Comparison between singly and repeatedly infected by two-way ANOVA p=0.0004) **c.** LT contributes to growth impairment on repeated challenge. Shown are mice (n=10/group) repeatedly challenged with either H10407 or the LT deletion mutant jf571. Stepped lines connect geometric mean values. Solid lines indicate linear regression. (Comparison between WT and ΔLT by two-way ANOVA p=0.0078).

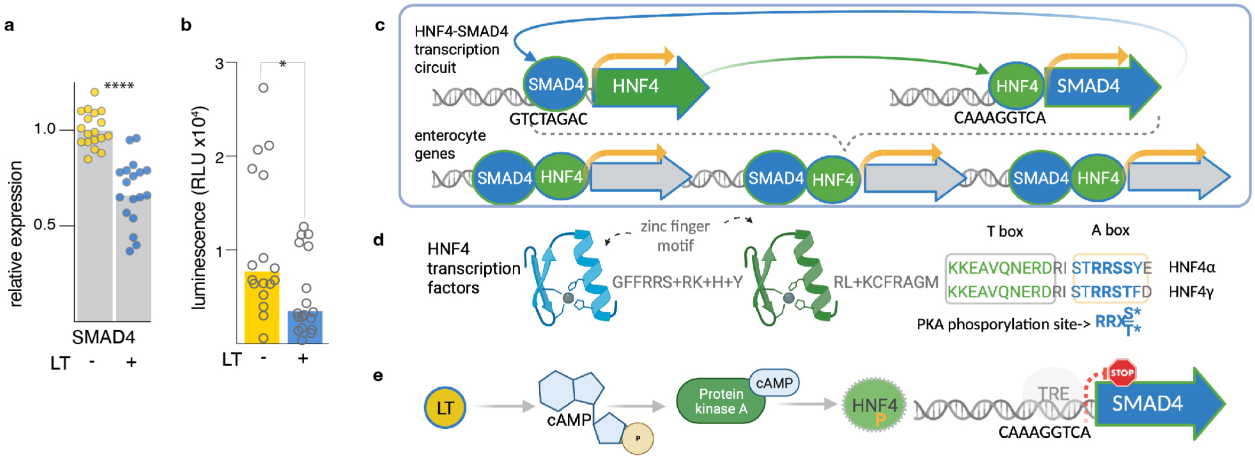

Supplementary figure 6. Potential mechanism underlying LT mediated disruption of brush border development. LT interrupts HNF4-SMAD4 mediated transcription. **a.** treatment of human-derived small intestinal enteroids (Hu235D) with LT leads to decreased SMAD4 transcription. Shown are TaqMan probe results from two independent experiments of polarized epithelial monolayers treated with LT. n=3 biological replicates x 3 technical replicates/ experiment =18 data points. ****p<0.0001 by Mann Whitney, two-tailed nonparametric comparisons to untreated cells. **b.** SMAD4-mediated transcription is decreased by LT. Shown are the combined results of three independent experiments in which Caco-2 cells transiently transfected with the SBE4-Luc plasmid containing 4 copies of the SMAD4 transcriptional response element were treated with LT (100 µg/ml x 16 hours). *=0.015 (Mann-Whitney, two-tailed). **c.** depiction of the HNF4-SMAD4 reciprocal transcription factor activation in which HNF4 activates transcription of SMAD4 and *vice versa* as (Chen, *et* al[1]). **d.** PKA-mediated phosphorylation of HNF4 paralogs interrupts a DNA-binding module (Viollet, *et al*[2].). **e.** Summary of proposed mechanism by which LT leads to depletion of transcription factors essential for enterocyte differentiation. LT-mediated activation of PKA leads to phosphorylation of HNF4 rendering these transcription factors incapable of binding to *cis* transcriptional response elements (TRE) in promotors of target genes including SMAD4. Decreased SMAD4 transcription in turn depletes available HNF4.

Supplementary Tables

supplementary table 1. strains and plasmids used in these studies

bacterial strains

strain designation	description	source
H10407	wild type ETEC strain, *eltAB*, *estP*, *estH*	[3]
jf570	Δ *eltAB* derivative of H10407	[4]
jf876	*lacZYA*::Km^R derivative of H10407	[4]
jf4763	*eltAB*::Sp^R, estP::Km^R, estH::Cm^R toxin-negative derivative of H10407.	this study

plasmids

designation	description	source
TR104	6 copies of HNF4 transcriptional response element upstream of Gaussia luciferase reporter (Gaussia-Dura Luc). Km^R, Pm^R	GeneCopoeia
SEAP-PA01	secreted alkaline phosphatase; CMV promotor; Amp^R	GeneCopoeia
SBE4-Luc	Luciferase reporter containing four copies of the SMAD4 binding site. 5'-GTCTAGAC-3'	addgene [5]

Sp^R spectinomycin resistant; Km^R kanamycin resistant; Cm^R chloramphenicol resistant; Pm^R puromycin resistant, Amp^R ampicillin resistant

**supplementary table 2 primers and probes for gene expression**			
RT-PCR primers			
gene	species	primer sequence (5'-3')	
		forward	reverse
SLC19A3	Human	TTCCTGGATTTACCCCACTG	TATGTCCAAACGGGGAAGA
VIL1	Human	CTGAGCGCCCAAGTCAAAG	AGCAGTCACCATCGAAGAAGC
MYO1A	Human	ACAGGCGAGAGTGGATCAG	GCACTGGGTTAGACTGTAGCAG
MYO7B	Human	TGGACGCCTCAGACGTGAT	CTGGTGACAAATTCCCCTCGG
SP-1	Human	CCATACCCCTTAACCCCG	GAATTTTCACTAATGTTTCCCACC
GAPDH	Human	CATTTCCTGGTATGACAACGA	CTTCCTCTTGTGCTCTTGCT
b-actin	Human	CATCCTGCGTCTGGACCT	TAATGTCACGCACGATTTCC
Ezr	Mouse	CAATCAACGTCCGGGTGAC	GCCAATCGTCTTTACCACCTGA
Vil1	Mouse	TCAAAGGCTCTCTCAACATCAC	AGCAGTCACCATCGAAGAAGC
Espn	Mouse	GGAAATACAGAGGGCGAAAGA	AGAAGCCGAGAGACTCCTATTA
Cdhr2	Mouse	GGCCTCGATTCTACAGCCAAT	GCTCAGAGCCATTCTCAGTCAC
Myo7B	Mouse	AGGGGACCTGAATGAGGCT	GCGGCTGTAGTAGATTTGTACCT
Ebp50	Mouse	AGGGTCCAAATGGCTACGG	CTCCACCAATCGGTCTCCAG
GAPDH	Mouse	AGGTCGGTGTGAACGGATTTG	TGTAGACCATGTAGTTGAGGTCA
TaqMan probes			
gene	species	chromosome	location
HNF4α	Human	20	44355801 - 44432845
HNF4γ	Human	8	75407567 - 75566834
SMAD4	Human	18	51030213 - 51085042

**supplementary table 3. differential gene expression in gastrointestinal cells following treatment with LT**			
response to LT	dataset	genes (n) $p<0.05$*	genes (n) $p<10^{-5}$*
higher	Caco-2	5,631	3,832
	ileal enteroids	2138	746
	both†	877	268
lower	Caco-2	5,263	3,687
	ileal enteroids	1,839	561
	both†	1,013	311

*differentially expressed genes identified by DESeq2[6]
† overlap in up- and downregulated genes was statistically higher than expected by random selection ($p<10^{-10}$, binomial distribution testing)
Expression values of LT-treated enteroid cells are relative to untreated cells; LT-treated Caco-2 values are relative to untreated Caco-2 cells plus those treated with mLT.

supplementary table 4. Transcription factor target sequence enrichment among genes differentially regulated in both RNA-seq datasets

	transcription factor target				
transcription factor	target designation	target motif	Total pathway size	Number of significant genes	FDR-adjusted P value
	*877 genes upregulated by LT in both RNA-seq datasets**				
AP1 / JUN	TGANTCA_V$AP1_C	TGANTCA	1108	91	4.2E-03
LEF1	CTTTGT_V$LEF1_Q2	CTTTGT	1939	142	4.2E-03
USF	V$USF_C	NCACGTGN	278	32	5.2E-03
	*1,013 genes downregulated by LT in both RNA-seq datasets**				
HNF4αγ	V$HNF4_01	NNNRGGNCAAAGKTCANNN	269	37	1.9E-04
HNF4αγ	V$HNF4_01_B	NRGGNCAAAGGTCAN	249	32	2.6E-03
USF	V$USF_02	NNRNCACGTGNYNN	270	32	8.8E-03
SREBP1	TCANNTGAY_V$SREBP1_01	TCANNTGAY	469	47	9.9E-03
NR2F2/COUP	V$COUP_01	TGAMCTTTGMMCYT	260	30	0.015
IRF1	STTTCRNTTT_V$IRF_Q6	STTTCRNTTT	185	23	0.025
USF	V$USF_01	NNRYCACGTGRYNN	254	28	0.038
HNF1	RGTTAMWNATT_V$HNF1_01	RGTTAMWNATT	72	12	0.044

*differentially expressed genes identified by DESeq2[6] $p<0.05$

**supplementary table 5. antibodies used in these studies**		
Specificity	description	source/reference
villin	Villin antibody (BDID2C3), mouse monoclonal IgG1	Santa Cruz sc-66022
mouse IgG	Goat anti-Mouse (H&L) IgG, highly cross-adsorbed antibody conjugated to Alexa Fluor 488	ThermoFisher A-11029
mouse IgA	Pre-absorbed, affinity purified goat anti-mouse IgA conjugated to HRP	Santa Cruz sc-3793
mouse IgG	Affinity purified horse anti-mouse IgG conjugated to HRP	Cell Signaling 7076
HNF4γ	rabbit polyclonal IgG against residues 327-408 of human HNF4γ	ThermoFisher 25801-1-AP
HNF4α	monoclonal IgG2a clone K9218) vs Baculovirus-expressed recombinant human HNF4α (aa 3-49)	ThermoFisher MA1-199
SP-1	rabbit monoclonal against synthetic peptide within Human SP1 aa 550-650.	abcam ab124804
SLC19A3	rabbit polyclonal IgG vs SLC19A3	proteintech 13407-1-AP
β-actin	β-Actin (C4) is a mouse monoclonal antibody (IgG1 kappa light chain) raised against gizzard Actin of chicken origin.	Santa Cruz sc-47778
rabbit IgG	IRDye® 800CW Goat anti-Rabbit IgG Secondary Antibody	LiCor 926-32211
mouse IgG	IRDye® 680LT Goat anti-Mouse IgG Secondary Antibody	LiCor 926-68020

**supplementary table 6**	
source data and links to original images	
item	url/DOI
figure 4b	https://doi.org/10.6084/m9.figshare.19251848.v1
figure 4c	https://doi.org/10.6084/m9.figshare.19251845.v1
figure 5b	https://doi.org/10.6084/m9.figshare.19287353.v1
figure 5e	https://doi.org/10.6084/m9.figshare.19251911.v1
figure 5g	https://doi.org/10.6084/m9.figshare.19251926.v1
figure 6c	https://doi.org/10.6084/m9.figshare.19251938.v1
figure 7c	https://doi.org/10.6084/m9.figshare.19742413.v1
supplementary figure 2	https://doi.org/10.6084/m9.figshare.21217775.v1
supplementary figure 3a	https://doi.org/10.6084/m9.figshare.21217721.v1
supplementary figure 3b	https://doi.org/10.6084/m9.figshare.21217712.v1
source data (xlsx file)	https://doi.org/10.6084/m9.figshare.21405237

1 Chen, L. *et al.* A reinforcing HNF4-SMAD4 feed-forward module stabilizes enterocyte identity. *Nat Genet* **51**, 777-785 (2019). https://doi.org:10.1038/s41588-019-0384-0

2 Viollet, B., Kahn, A. & Raymondjean, M. Protein kinase A-dependent phosphorylation modulates DNA-binding activity of hepatocyte nuclear factor 4. *Molecular and cellular biology* **17**, 4208-4219 (1997). https://doi.org:10.1128/MCB.17.8.4208

3 Evans, D. J., Jr. & Evans, D. G. Three characteristics associated with enterotoxigenic Escherichia coli isolated from man. *Infect Immun* **8**, 322-328 (1973). https://doi.org:DOI: 10.1128/iai.8.3.322-328.1973

4 Dorsey, F. C., Fischer, J. F. & Fleckenstein, J. M. Directed delivery of heat-labile enterotoxin by enterotoxigenic Escherichia coli. *Cell Microbiol* **8**, 1516-1527 (2006). https://doi.org:10.1111/j.1462-5822.2006.00736.x

5 Zawel, L. *et al.* Human Smad3 and Smad4 are sequence-specific transcription activators. *Mol Cell* **1**, 611-617 (1998). https://doi.org:10.1016/s1097-2765(00)80061-1

6 Love, M. I., Huber, W. & Anders, S. Moderated estimation of fold change and dispersion for RNA-seq data with DESeq2. *Genome Biol* **15**, 550 (2014). https://doi.org:10.1186/s13059-014-0550-8
