# Supplementary material for: Enterotoxigenic Escherichia coli heat-labile toxin drives enteropathic changes in small intestinal epithelia
Source: Nat Commun. 2022 Nov 12;13:6886. doi: 10.1038/s41467-022-34687-7 (PMC9653437; doi:10.1038/s41467-022-34687-7)
Supplement: Supplementary file 3 — Description of Additional Supplementary Files [file 41467_2022_34687_MOESM3_ESM.pdf]

Description of Additional Supplementary Files

File Name: Supplementary Data 1
Description: Gene expression profiles

File Name: Supplementary Data 2
Description: Gene ontology profiles
