# Supplementary material for: Enterotoxigenic Escherichia coli heat-labile toxin drives enteropathic changes in small intestinal epithelia
Source: Nat Commun. 2022 Nov 12;13:6886. doi: 10.1038/s41467-022-34687-7 (PMC9653437; doi:10.1038/s41467-022-34687-7)
Supplement: Supplementary file 6 — Reporting Summary [file 41467_2022_34687_MOESM6_ESM.pdf]

nature portfolio

Corresponding author(s): James M. Fleckenstein

Last updated by author(s): Oct 27, 2022

Reporting Summary

Nature Portfolio wishes to improve the reproducibility of the work that we publish. This form provides structure for consistency and transparency in reporting. For further information on Nature Portfolio policies, see our Editorial Policies and the Editorial Policy Checklist.

Statistics

For all statistical analyses, confirm that the following items are present in the figure legend, table legend, main text, or Methods section.

n/a	Confirmed	
☐	☒	The exact sample size (*n*) for each experimental group/condition, given as a discrete number and unit of measurement
☐	☒	A statement on whether measurements were taken from distinct samples or whether the same sample was measured repeatedly
☐	☒	The statistical test(s) used AND whether they are one- or two-sided<br>*Only common tests should be described solely by name; describe more complex techniques in the Methods section.*
☒	☐	A description of all covariates tested
☐	☒	A description of any assumptions or corrections, such as tests of normality and adjustment for multiple comparisons
☐	☒	A full description of the statistical parameters including central tendency (e.g. means) or other basic estimates (e.g. regression coefficient) AND variation (e.g. standard deviation) or associated estimates of uncertainty (e.g. confidence intervals)
☐	☒	For null hypothesis testing, the test statistic (e.g. *F*, *t*, *r*) with confidence intervals, effect sizes, degrees of freedom and *P* value noted<br>*Give P values as exact values whenever suitable.*
☒	☐	For Bayesian analysis, information on the choice of priors and Markov chain Monte Carlo settings
☒	☐	For hierarchical and complex designs, identification of the appropriate level for tests and full reporting of outcomes
☒	☐	Estimates of effect sizes (e.g. Cohen's *d*, Pearson's *r*), indicating how they were calculated

Our web collection on statistics for biologists contains articles on many of the points above.

Software and code

Policy information about availability of computer code

Data collection	routine data collection was performed in Microsoft Excel v16.66.1
Data analysis	DESeq2 was used in gene expression analysis of RNAseq data. Microsoft Excel v16.66.1 was used in heatmap and bar graph visualization. Sailfish v0.6.3 was used to quantify transcript counts. R/Bioconductor packages GAGE and Pathwiew were used in Gene ontology analysis. Ontology-independent analysis was performed with CompBio v1.4.

For manuscripts utilizing custom algorithms or software that are central to the research but not yet described in published literature, software must be made available to editors and reviewers. We strongly encourage code deposition in a community repository (e.g. GitHub). See the Nature Portfolio guidelines for submitting code & software for further information.

Data

Policy information about availability of data

All manuscripts must include a data availability statement. This statement should provide the following information, where applicable:

- Accession codes, unique identifiers, or web links for publicly available datasets
- A description of any restrictions on data availability
- For clinical datasets or third party data, please ensure that the statement adheres to our policy

Provide your data availability statement here.

Human research participants

Policy information about studies involving human research participants and Sex and Gender in Research.

Reporting on sex and gender	*Use the terms sex (biological attribute) and gender (shaped by social and cultural circumstances) carefully in order to avoid confusing both terms. Indicate if findings apply to only one sex or gender; describe whether sex and gender were considered in study design whether sex and/or gender was determined based on self-reporting or assigned and methods used. Provide in the source data disaggregated sex and gender data where this information has been collected, and consent has been obtained for sharing of individual-level data; provide overall numbers in this Reporting Summary. Please state if this information has not been collected. Report sex- and gender-based analyses where performed, justify reasons for lack of sex- and gender-based analysis.*
Population characteristics	*Describe the covariate-relevant population characteristics of the human research participants (e.g. age, genotypic information, past and current diagnosis and treatment categories). If you filled out the behavioural & social sciences study design questions and have nothing to add here, write "See above."*
Recruitment	*Describe how participants were recruited. Outline any potential self-selection bias or other biases that may be present and how these are likely to impact results.*
Ethics oversight	*Identify the organization(s) that approved the study protocol.*

Note that full information on the approval of the study protocol must also be provided in the manuscript.

Field-specific reporting

Please select the one below that is the best fit for your research. If you are not sure, read the appropriate sections before making your selection.

☒ Life sciences ☐ Behavioural & social sciences ☐ Ecological, evolutionary & environmental sciences

For a reference copy of the document with all sections, see nature.com/documents/nr-reporting-summary-flat.pdf

Life sciences study design

All studies must disclose on these points even when the disclosure is negative.

Sample size	no a priori sample size determination was performed; sample sizes were determined empirically from prior experiments.
Data exclusions	no data were excluded from the analysis.
Replication	data on experimental and technical replicates are included in the legend of each corresponding figure.
Randomization	study design with neonatal mice precluded randomization of animals
Blinding	study design precluded blinding of the limited number of investigators involved in the different arms of the data collection.

Reporting for specific materials, systems and methods

We require information from authors about some types of materials, experimental systems and methods used in many studies. Here, indicate whether each material, system or method listed is relevant to your study. If you are not sure if a list item applies to your research, read the appropriate section before selecting a response.

Materials & experimental systems

n/a	Involved in the study	
☐	☐	Antibodies
☐	☒	Eukaryotic cell lines
☒	☐	Palaeontology and archaeology
☐	☒	Animals and other organisms
☒	☐	Clinical data
☒	☐	Dual use research of concern

Methods

n/a	Involved in the study	
☒	☐	ChIP-seq
☒	☐	Flow cytometry
☒	☐	MRI-based neuroimaging

Antibodies

Antibodies used	provided in supplementary table 5
Validation	validation statements are provided for individual antibodies on the manufacturer's websites.

Eukaryotic cell lines

Policy information about cell lines and Sex and Gender in Research

Cell line source(s)	cell line Hu235D from human small intestine was obtained from the Digestive disease Research Core Center at Washington University School of Medicine. Caco-2 cells were obtained from ATCC
Authentication	Both Caco-2 and Hu235D lines were tested for their response to LT by cAMP ELISA prior to initiation of the studies outlined here
Mycoplasma contamination	mycoplasma screening of cell lines indicated that both Hu235D and Caco-2 cells were free of mycoplasma contamination
Commonly misidentified lines (See ICLAC register)	no commonly misidentified cell lines were used in these studies

Animals and other research organisms

Policy information about studies involving animals; ARRIVE guidelines recommended for reporting animal research, and Sex and Gender in Research

Laboratory animals	CD-1 male and female mice obtained from Charles River served as the parental strain in all experiments involving neonatal mice. Neonates were 3 days of age on challenge.
Wild animals	these studies did not involve wild animals
Reporting on sex	equal numbers of male and female mice were employed throughout the studies involving neonatal mice.
Field-collected samples	the study did not involve samples collected from the field.
Ethics oversight	The studies reported here comply with all relevant ethical regulations and the study protocols have been approved by the IACUC, Institutional Biosafety, and Institutional Review Boards at Washington University in Saint Louis School of Medicine.

Note that full information on the approval of the study protocol must also be provided in the manuscript.
